# The care pathway for hip fracture from acute phase to rehabilitation

**DOI:** 10.1186/1472-6963-14-S2-P74

**Published:** 2014-07-07

**Authors:** Paolo Sciattella, Valeria Belleudi, Paola Colais, Mirko Di Martino, Luigi Pinnarelli, Francesca Mataloni, Marina Davoli, Danilo Fusco

**Affiliations:** 1 Department of Epidemiology, Lazio Region Health Service, Rome, Italy

## Background

Hip fractures are the leading cause of hospitalization for injuries among the elderly population and have a substantial impact on both the patient and the healthcare system. The care pathway for a person with a disability goes through a set of integrated and multidisciplinary activities and care interventions. Starting with timely surgery in the acute phase, followed by adequate rehabilitation assistance and ending with territorial assistance.

## Aims

The aim of the study is to evaluate the association between early surgery and access to rehabilitation of elderly patients with hip fracture.

## Materials and methods

We identified elderly patients hospitalised for hip fracture, between 1 January 2012 and 31 October 2012 using Hospital Information System (HIS) of the Lazio region. The outcome considered was the access to rehabilitation within 60 days from the date of hospital discharge. Rehabilitation access was derived from Admission/Discharge Rehabilitation Report (ADRR), for hospital rehabilitation, and from Residential Rehabilitation Information System (RRIS).

We considered clinical variables, residence and level of education as potential risk factors of the outcome. The factors significantly associated with outcome (gender, age, comorbidities, residence, level of education, hospital stay) were selected by a bootstrap stepwise procedure and their Risks Ratio (RR) were estimated through a multivariate regression model.

## Results

We selected 5,030 patients aged 65+ hospitalised for hip fracture in Lazio region, 59% with an access to rehabilitative care within 60 days from discharge. The access to rehabilitation was less likely for older patients (adjusted RR=0.74, p<0.001), for patients with a longer acute event hospital stay (adjusted RR=0.74, p<0.001) and for patients with senile dementia (adjusted RR=0.66, p<0.001). By contrast, the probability was higher for patient who had surgery within 48h (adjusted RR=2.60, p<0.001) and for residents in Rome (adjusted RR=1.24p<0.001).

The level of education seems to be negatively associated with the outcome (adjusted RR=0.79 p<0.05 for patients with high level), probably because patients with a higher level of education could more easily obtain access to private services.

## Conclusions

Despite the fact that following hip fracture rehabilitation should involve all patients, in the Lazio region the percentage of access to rehabilitation within 60 days from hospital discharge is 59%. Access to rehabilitation is strongly dependent on age, clinical characteristics and acute hospital care; in particular, waiting time for surgical treatment is strongly correlated with access to rehabilitation. This could suggest that the care pathway evaluation should take into account the association between the acute and rehabilitation phase.

